# Unravelling Migraine Stigma: A Comprehensive Review of Its Impact and Strategies for Change

**DOI:** 10.3390/jcm13175222

**Published:** 2024-09-03

**Authors:** Javier Casas-Limón, Sonia Quintas, Alba López-Bravo, Alicia Alpuente, Alberto Andrés-López, María Victoria Castro-Sánchez, Javier Amós Membrilla, Cristian Morales-Hernández, Nuria González-García, Pablo Irimia

**Affiliations:** 1 Headache Unit, Hospital Universitario Fundación Alcorcón, 28922 Alcorcón, Spain; jcasaslimon@gmail.com; 2 Headache Unit, Hospital Universitario de la Princesa, Instituto de Investigación Sanitaria Princesa (IIS-Princesa), 28006 Madrid, Spain; sonia.qg@gmail.com; 3 Headache Unit, Hospital Reina Sofía, 31500 Tudela, Spain; alba.lopez.bravo@gmail.com; 4 Headache Unit, Hospital Universitario Vall d’Hebron, 08035 Barcelona, Spain; alpuente1920@gmail.com; 5 Headache Unit, Complejo Hospitalario Universitario de Albacete, 02006 Albacete, Spain; albertoanlo91@gmail.com; 6 Headache Unit, Hospital Regional Universitario de Málaga, 29002 Málaga, Spain; mvictoriacastrosanchez@gmail.com; 7 Headache Unit, Hospital Comarcal Francesc de Borja, 46702 Gandía, Spain; membrillaja@gmail.com; 8 Headache Unit, Hospital Universitario de Canarias, 38320 La Laguna, Spain; andariell2010@gmail.com; 9 Headache Unit, Hospital Universitario Clínico San Carlos, 28040 Madrid, Spain; nurigongar@gmail.com; 10 Headache Unit, Clínica Universitaria de Navarra, 31008 Pamplona, Spain

**Keywords:** migraine, stigma, chronic disease, quality of life, mental health, prejudice, health policy, patient advocacy, health education, discrimination

## Abstract

Migraine-related stigma is a pervasive issue impacting nearly half of chronic migraine patients, with significant consequences for their quality of life, disability and mental health. Despite its profound effects, migraine stigma remains under-recognised in both clinical practice and research. This narrative review explores the three primary types of stigmas affecting migraine patients: public, structural and internalised. Public stigma involves negative societal attitudes and stereotypes that trivialise the condition. Structural stigma is reflected in policies that restrict access to necessary care and resources. Internalised stigma occurs when patients absorb these negative views, leading to self-blame and diminished self-worth. Addressing these different types of stigmas is crucial for improving the understanding, diagnosis and treatment of migraine. Educational efforts, advocacy and policy reform are essential strategies in this context. A deep understanding of stigma is vital for developing effective interventions that enhance clinical management and patient quality of life. Ultimately, reducing stigma can lead to better health outcomes and a more comprehensive approach to migraine care.

## 1. Introduction

Migraine is one of the most common, disabling and highly stigmatised neurological disorders [1,2,3,4,5]. The estimated prevalence of episodic migraine and chronic migraine in the general population is 12% and 1.4–2.2%, respectively [1,6]. According to the Global Burden of Disease study, migraine is the third leading cause of disability, particularly affecting middle-aged women, with peak impact and prevalence occurring between 20 and 55 years, the most productive years of a person’s life [2,7,8].

Migraine is associated with a significant economic and social burden, a wide range of comorbidities (particularly psychiatric) and a diminished health-related quality of life [1,2,9]. The number of monthly migraine days is the key factor influencing disability, cost, quality of life and increased psychiatric comorbidity [8,9,10,11,12,13].

The pathophysiology of migraine is not completely elucidated [14,15]. Genetic studies have identified 123 susceptibility loci, confirming its polygenic nature [16], with rare monogenic forms like familial hemiplegic migraine [17]. The trigeminovascular system is central to migraine pain, which is initiated by the activation and sensitisation of first-order neurons in the trigeminal ganglion [18,19]. These neurons innervate the meninges and transmit nociceptive signals to the brainstem, where second-order neurons are activated. The signals are then relayed to third-order neurons in the thalamus, which ultimately project to the somatosensory cortex, giving rise to pain perception [14,15,18,19]. Trigeminal activation mediates migraine pain through neuropeptides such as calcitonin gene-related peptide (CGRP) and pituitary adenylate cyclase-activating polypeptide (PACAP) released in the dura mater [20,21]. Additionally, cortical spreading depression, a phenomenon that explains the migraine aura, can activate trigeminovascular neurons, further contributing to migraine pathophysiology [14,15,18,19].

Recent years have witnessed a growing interest in the factors that influence disability, quality of life and economic burden in patients with migraine [2,11,13,22]; however, migraine-related stigma has been largely overlooked [23]. Historically, mental illnesses have been the most stigmatised, leading to a disproportionate focus on these conditions in most of the stigma research conducted to date [3,23,24]. Emerging evidence suggests that a substantial proportion of migraine patients experience stigma, with prevalence rates ranging from 25.5% among those with fewer than 4 monthly headache days to 47.5% among those with 15 or more headache days per month. This stigma not only exacerbates disability and diminishes the quality of life [25] but is also closely associated with the presence of psychiatric symptoms [26]. Addressing this overlooked aspect is crucial, as understanding and mitigating stigma can substantially improve patient outcomes and well-being.

In this work, we conducted a comprehensive narrative review to examine the far-reaching consequences of stigma on migraine patients, including its impact on access to timely diagnosis and treatment, its detrimental effects on disability and quality of life and its association with prejudice, discrimination and social isolation. We also explore strategies to address migraine-related stigma.

## 2. Stigma: Definitions and Types

The term stigma was originally described as a physical mark or brand used in ancient Greece to identify slaves, distinguishing them from free individuals. While the term stigmata is also used to describe wounds resembling those of Christ’s crucifixion, in modern usage, stigma generally refers to a mark of shame, a disgrace or a defect [24]. In the health context, stigma is defined by the World Health Organization (WHO) as ‘a mark of shame, disgrace or disapproval that results in an individual being rejected, discriminated against and excluded from participating in a number of different areas of society’ [27]. Stigma arises from identifying and labelling differences associated with a disease. These differences are often linked to negative attributes, leading to divisions between the sick and the healthy. Consequently, individuals stigmatised by the disease may feel that their illness is not understood and may experience loss of status, discrimination and social isolation [28,29].

Sociological research on stigma has identified three primary types of stigma (Table 1): public, structural and internalised [29,30]. Public stigma refers to the widespread stereotypes and negative attitudes that society holds towards people with migraine, often manifesting in hurtful comments such as ‘It’s just a headache’, ‘You can fix this by improving your lifestyle’ or ‘This headache thing is a typical excuse to skip work’. Structural stigma occurs when public stigma is perpetuated and reinforced through institutional policies and practices, resulting in systematic barriers that intentionally or unintentionally limit opportunities for individuals with migraines. This can manifest in many ways, such as inadequate funding for migraine care or research and restrictive access to sick leave or disability benefits for migraines. These structural barriers can further marginalise individuals with migraine and hinder their access to necessary resources and support. Finally, internalised stigma occurs when people affected by stigma begin to recognise and believe the negative stereotypes about their disease. Some patients may internalise these beliefs and consider themselves ‘incompetent’ or ‘unable to assume responsibilities at work’.

The three types of stigmas are intricately interconnected. When society lacks understanding and perpetuates negative stereotypes about migraine, it can lead to a lack of prioritisation from healthcare policymakers and inadequate support for those affected. Eventually, individuals with migraine may internalise these negative stereotypes, leading to decreased self-esteem and quality of life. This interplay between public misconception, structural neglect and internalised shame exacerbates the burden of migraine.

## 3. Gender Stereotypes and Migraine-Related Stigma

Gender stereotypes surrounding migraine have persisted since at least the 18th century, perpetuating stigma against patients [23]. Historically, migraine has been misconstrued as a quintessentially female condition, associated with ‘women of fashion’, ‘young female martyrs’, and ‘mothers in the lower classes’. This led to the erroneous notion that women’s brains were ‘fragile’ and prone to ’overload´, implying they had a diminished capacity for intellectual work or to assume responsibilities. Additionally, the higher prevalence of migraine in women has led to misconceptions about its aetiology, with some attributing it to psychological factors, which has contributed to doubts about its legitimacy as a disease and perpetuated gender-based labels like ‘neurotic housewives’ [23,31]. Notably, Harold George Wolff (28 May 1898–21 February 1962), considered the father of modern headache research, inadvertently reinforced these stereotypes [31,32]. In his analysis of patients, Wolff often portrayed his male patients in a positive light, describing them as driven, capable, meticulous and successful. He described these men were overexerting themselves and suggested they take time to relax and engage in more physical activity. In contrast, his female patients were described through a more critical lens, being labelled as lacking, unsatisfied and emotionally cold. Their migraines, unlike those of men, were interpreted as being rooted in psychological issues.

Migraine affects women disproportionately, with a higher prevalence, severity and duration of pain [33]. While hormonal factors primarily contribute to these differences, a persistent perception remains that women’s greater representation in outpatient clinics for headache treatment may be due to their greater cultural permission to express pain openly, whereas men with chronic pain have traditionally been perceived as less masculine [34]. Furthermore, entrenched gender stereotypes suggest that men are inherently better at enduring severe pain and are more resilient than women [34,35].

Finally, the historical predominance of male physicians has contributed to a systemic bias, leading to women being taken less seriously when seeking medical care for chronic pain [34]. Consequently, women are often unfairly stereotyped as hysterical and overly complaining, perpetuating the misconception that their pain is psychological rather than somatic and that they are unwilling to recover [32,34,35]. Furthermore, the perception of sexism-related stigma by female patients exacerbates their subjective experience of pain, creating a vicious cycle [34,35,36].

## 4. Cultural Influence on Migraine-Related Stigma

Culturally rooted health beliefs shape perceptions of the underlying causes of the disease, influence treatment acceptance and contribute to stigma. These beliefs have been slightly more explored in psychiatric disorders and chronic pain [37,38,39,40]. In the context of pain, a person’s culture determines how pain is perceived, experienced and communicated. A few studies have analysed the impact of cultural beliefs on the stigma faced by individuals with headaches. In rural African communities, headaches are often attributed to supernatural causes, such as evil spirits or curses, leading to stigmatisation and delaying effective treatments [41]. Furthermore, religious beliefs influence pain perception, management and coping strategies [42,43]. Among migraine patients, prayer is considered helpful in reducing the intensity of attacks [44]. Thus, culturally sensitive approaches are crucial for effective headache management, as understanding the cultural context of pain is essential for building strong physician–patient relationships and optimising treatment outcomes.

## 5. Stigma Hinders Migraine Care

Migraine diagnosis is based on clinical assessment using the International Classification of Headache Disorders criteria [4]. However, the absence of diagnostic biomarkers, combined with normal neuroimaging results, often contributes to migraine being unrecognised and misunderstood by society and the medical and scientific communities [45,46]. This lack of recognition renders migraine a socially invisible disease, vulnerable to stigma [23,47,48].

Migraine remains a significantly underdiagnosed and undertreated condition worldwide [49,50,51,52]. A recent population-based study in Spain revealed that only 56.2% of patients had received a confirmed diagnosis of migraine from a healthcare professional [52]. Furthermore, approximately 30% of survey respondents were reluctant to seek medical attention for their migraine symptoms [52]. Internalised stigma appears to be a contributing factor to this hesitancy. The most frequent reason for not seeking consultation was the belief that their symptoms were ‘not serious/painful enough’, reported by 9.1% of patients. Other reasons included reliance on over-the-counter medications or self-management (7.0%), desire to handle the condition independently (6.9%) and fear of not being taken seriously by healthcare professionals (6.6%).

A major obstacle to an accurate diagnosis is the inadequate training of doctors who care for patients with migraine [53]. According to the World Health Organization’s Atlas of Headache Disorders and Resources in the World, developed in collaboration with Lifting The Burden, a survey of 101 countries revealed that 75% identified professional education as the top priority for improving migraine care [54]. Despite its high prevalence, training on this disease is inadequate in medical schools and specialised neurology training programmes. This issue is concerning in high-income countries [55,56,57] but even more worrisome in low-income nations [58]. Concerning medical management, triptans are not extensively used in patients diagnosed with migraine and preventive medication is used by only 36% of eligible individuals [52]. This treatment gap underscores the need for enhanced education and more effective implementation of treatment guidelines in clinical practice [59].

## 6. Structural Stigma in Migraine

Migraine research has historically received insufficient funding from the National Institutes of Health (NIH), despite its high prevalence and significant societal impact [29,60,61,62]. This funding disparity perpetuates the stigmatisation of migraine and is striking when compared with other highly prevalent and disabling neurological diseases like Alzheimer’s disease. While Alzheimer’s research receives over USD 3 billion annually, migraine research is allocated only about USD 13 million [48,61,62]. The funding disparity becomes even more striking when examining per capita spending. In 2007, NIH funding per disability-adjusted life-year was a mere USD 13 for migraine, compared with USD 739 for epilepsy and USD 232 for stroke [48,61,62]. On a per-person-with-disorder basis, an individual with migraine received only USD 0.36, while stroke patients received USD 48.57 and epilepsy patients received USD 35.15 [48,61,62]. Similarly, Alzheimer’s disease currently receives approximately USD 239 per person affected. These figures not only highlight the scarcity of funding for migraine research but also reflect a broader lack of recognition of the social and economic impact of migraine.

In 2019, only 55 NIH extramural principal investigators received funding for ‘headache’ research grants, in stark contrast to the average of 322 (±84) funded investigators for other chronic neurological diseases, such as Parkinson’s disease, epilepsy and multiple sclerosis [48,61,62]. This discrepancy illustrates the limited academic and research focus on migraine compared with other neurological conditions. Moreover, this lack of financial support has direct consequences, driving neurologists and researchers to redirect their focus towards better-funded areas. This trend may contribute to a decrease in the number of innovative studies, ultimately hindering progress in understanding migraine and developing new more effective treatments. To address this issue, it is crucial to equitably increase funding.

Laws are a particularly powerful mediator for structural stigma. The current legal framework fails to adequately support migraine patients [29,63]. In many countries, obtaining sick leave or disability benefits for migraines is a significant challenge. The process of securing long-term disability benefits can be lengthy, taking up to three years, and has a low success rate, with less than 20% of headache-related claims being approved [29,64]. This lack of legislative support exacerbates the burden on migraine patients and contributes to the social trivialisation of migraines.

## 7. Quantifying Migraine Stigma: Tools and Insights

Stigma is a significant concern in chronic health conditions, and it can be measured using standardised scales. In migraine research, two primary scales have been used to capture stigma: the stigma scale for chronic illness (SSCI) and the migraine-related stigma (MiRS) questionnaire [25,65,66]. These tools are crucial for comparing stigma levels across different diseases and quantifying it. This quantification allows healthcare providers to tailor treatment strategies based on the degree of stigma and patient experiences. For instance, a higher stigma score on the SSCI or MiRS may indicate the need for a more comprehensive treatment approach, incorporating psychological support and social interventions.

The SSCI is a 24-item questionnaire designed to assess both internalised stigma (self-perception) and enacted stigma (how individuals are treated by others). It provides a quantitative measure of stigma, enabling systematic analysis and comparison across various neurological conditions, including migraine [65]. However, the SSCI is not suitable for use as a screening tool to differentiate between patients with and without stigma. A study of 511 patients with neurological disorders such as stroke, epilepsy, multiple sclerosis, Parkinson’s disease and motor neuron disease (but not migraine) reported an average stigma score of 42.7 (standard deviation = 19.7) [65]. In the study by Young et al. [5], SSCI was used to evaluate stigma in 123 episodic migraine patients, 123 chronic migraine patients and 62 epilepsy patients in a clinical setting. Stigma scores for epilepsy and episodic migraine aligned with those with other neurological conditions (average SSCI score: 42.7 ± 19.7). However, patients with chronic migraine reported significantly higher scores, averaging 54.0 (±20.2) [5]. Other studies comparing patients with episodic migraine, chronic migraine and epilepsy using the SSCI also revealed that the mean SSCI score was higher (51.6  ±  15.0) in the chronic migraine group than in the episodic migraine (45.0  ±  13.5) and epilepsy (47.6  ±  15.5) groups [67]. Conversely, two studies conducted in Turkey reported that patients with epilepsy experienced more stigma and that those with migraine primarily experienced enacted stigma [68,69]. These differences among studies were likely attributable to differences in cultural and religious backgrounds, where seizures are perceived as more externally stigmatising. A recent survey by the European Migraine & Headache Alliance used the SSCI scale to assess migraine stigma. Out of 4210 respondents, 57% reported eight or more migraine days per month. The survey highlighted that stigma is more prevalent in medical and workplace settings, with 74% feeling misunderstood by healthcare professionals and 79% reporting career impacts. Stigma was deemed more severe than for other neurological conditions, though less than mental health conditions [70].

The MiRS questionnaire was specifically developed to quantify stigma in individuals with migraine through a comprehensive review process involving existing stigma scales, expert input and focus group discussions with patients with migraine [25,66]. In a recent study using the MiRS questionnaire, approximately one-third (31.7%) of respondents reported frequently experiencing migraine-related stigma [25]. Notably, those with more frequent headaches (eight or more headache days per month) were more likely to experience stigma (in over 40% of cases) compared with those with less than four headache days (25.5% of cases). However, the study highlighted that even among those with less frequent headaches, stigma remained a significant concern, emphasising that frequency alone is insufficient for assessing stigma.

## 8. Neurologist’s Stigma towards Patients with Migraine

Despite recognising migraine as a neurobiological disease, many healthcare professionals, including neurologists, hold a notable stigma towards patients with migraine [71]. Furthermore, 82% of neurologists find treating migraine patients challenging and 67% report that it is emotionally draining [72,73]. This stigma is not only felt by healthcare providers but also by patients, who often feel dismissed and disbelieved by physicians regarding their pain complaints [74].

The shortage of headache specialists is another manifestation of stigma, reflecting both the preference of healthcare professionals to focus on other conditions and the tendency of healthcare policies to downplay the significance of migraine. Despite the high prevalence and increasing complexity of migraine treatment, the current number of practicing headache specialists is inadequate. A recent study suggested that the USA needs 3700 headache specialists to meet patient demand [75].

Moreover, the insufficient training of residents and other healthcare professionals further compounds the issue, leading to challenges in diagnosing and adequately treating migraine patients. Surveys conducted in Europe and the USA highlight the lack of mandatory headache rotations during neurology residency, with many residents reporting incomplete training and limited exposure to headache research [48,56]. This suboptimal training further perpetuates the stigma, as headache disorders are often viewed as having low prestige within the medical community [48,56,76,77,78,79].

## 9. Migraine and Stigma in the Workplace

Migraine, the leading cause of disability among individuals under 50, has a profound impact on professional life, striking during peak productivity years [2,80,81,82,83,84,85]. For this reason, workplace stigma among employers and co-workers is particularly significant. A survey of 2000 non-migraine employees conducted in the USA revealed the following alarming misconceptions: 31% of respondents believed that migraine sufferers use their condition to avoid work commitments and 27% thought they used it to garner attention [86]. Furthermore, nearly 45% of respondents felt that migraines should be easily treated and 36% believed that migraines resulted from unhealthy behaviour [86]. Notably, stigma was more pronounced among those acquainted with migraine sufferers, especially when they needed to take work absences due to their condition [87]. Another survey revealed that over half of managers did not consider headaches a valid reason for work absence, leading many migraine patients to avoid disclosing their condition due to stigma. Consequently, more than half of the patients who missed work due to migraines did not report it as the cause [88]. A recent study conducted at a Japanese information technology company with over 70,000 employees found that 81% of those who experienced headaches had never consulted a doctor. Notably, one of the most common reasons for not seeking help was the inability to take time off work [89].

The disabling nature of a migraine attack is undeniable. Patients report being 46% less effective at work during an attack, citing headache as the most disabling symptom, followed by difficulty thinking, photophobia, osmophobia and nausea [2,7,90]. Moreover, migraine’s impact on work productivity extends beyond non-headache days, as up to 40% of patients experience interictal symptoms like attention and executive function difficulties, processing speed issues and memory problems [91,92,93]. Some patients develop cogniphobia, a fear that mentally demanding tasks may trigger or worsen a migraine attack, leading to decreased confidence in their work abilities and impaired professional performance [94]. The unpredictability of migraine attacks also induces anxiety, leading patients to avoid meetings for fear of needing to leave suddenly, which significantly reduces their quality of life [12,95].

Another significant issue is that migraines can hinder professional advancement, with most patients experiencing career setbacks due to their condition. In a survey of 400 patients, 22% reported having to change careers due to migraines, limiting their opportunities for growth and leading to reduced socioeconomic status, decreased access to medical care, increased stress and worsening migraine symptoms [92]. To reduce workplace stigma, participants suggested increased awareness and understanding of the disease, followed by workplace support and flexibility. Notably, 85% of participants wanted their coworkers to recognise that migraine is a debilitating condition beyond ‘just a headache’ [92].

The economic impact of migraine-related productivity loss is substantial, primarily due to presenteeism—working while experiencing migraine symptoms, which reduces productivity [11,13]. Unlike absenteeism, there are no standardised methods for assessing the impact of presenteeism, but it is estimated to cost 3–10 times more than absenteeism [96], with costs increasing alongside headache frequency. In a survey conducted in Turkey, patients with chronic migraine reported an average of 3.5 days of absenteeism per year compared with 87 days of presenteeism, resulting in 38% reduced productivity annually [97]. Another Turkish study showed that migraine sufferers (2.5% of the workforce) accounted for over 45% of economic losses due to presenteeism [98]. A US-based study analysing the economic cost of presenteeism for 22 common diseases found that migraine was the second most costly, behind only allergies [87]. Extrapolating these data suggests that migraine is responsible for approximately 16% of workplace presenteeism, with an annual cost of approximately USD 240 billion [87]. The Fujitsu study reported that presenteeism and absenteeism due to migraines resulted in an average annual cost of approximately USD 2300 per patient [89]. In a Spanish study [13], reducing one migraine day per month leads to an average yearly savings of €744 per patient with episodic migraine and €663 per patient with chronic migraine. If the number of migraine days is reduced by 50%, the economic savings would be €2232 per patient with episodic migraine and €6632 per patient with chronic migraine annually [13].

Many of the reasons for presenteeism are linked to migraine-associated stigma. Migraine patients often report a significant lack of understanding of their condition in the workplace, impaired relationships with colleagues due to their headaches and feelings of guilt about burdening their bosses and coworkers [99,100].

### Supporting People with Migraine in the Workplace

Several strategies can be implemented to support patients with migraine and reduce stigma in the workplace, including education and awareness, creating a migraine-friendly environment and flexible work arrangements such as flexible working hours with options for remote work.

First, education and awareness are critical components. Workplace educational programmes have been shown to significantly increase productivity and decrease stigma among patients with migraine [70,95,101,102,103,104,105,106,107]. These programmes aim to raise awareness, improve workplace relationships and provide support through education [108]. For example, the Spanish Postal Service implemented an educational programme that included a survey to identify employees with headaches. Those diagnosed with migraine received health education and treatment, leading to a 53% reduction in work absences and a significant increase in productivity on days when employees experienced migraine attacks [108]. Studies conducted in other countries have corroborated that such interventions reduce absenteeism, improve productivity and decrease migraine-related costs [89,109,110,111,112]. Notably, these programmes not only reduce stigma but also enhance understanding and support from colleagues, which are key concerns for patients [89].

Second, creating a migraine-friendly work environment is crucial to support employees with migraine. Recent initiatives by the IHS-Global Patient Advocacy Coalition and the European Migraine & Headache Alliance aim to promote such environments. Simple adjustments such as using natural light, reducing noise, eliminating strong odours, providing access to water and services, offering regular breaks and improving air quality can significantly reduce disability and increase productivity among patients with migraine [100,101]. Additionally, avoiding excessive workload, long working hours and rigid schedules can also enhance productivity and quality of life, ultimately helping to reduce the stigma associated with migraine [64]. A case study of two chronic migraine patients revealed that working night and rotating shifts exacerbated their migraine frequency and disability, despite optimal headache management and treatment adherence. However, after switching to exclusively day shifts, their migraine patterns improved significantly, becoming less frequent and less disabling, leading to improved quality of life and productivity [102].

Finally, flexibility in the workplace is essential to support employees with migraine. Many patients with migraine are eager to work but may need to adapt their schedule due to acute attacks. Flexible work arrangements, such as allowing remote work or adjusting work hours, can help accommodate their needs and reduce internalised stigma. However, these measures are still limited in many workplaces and patients with migraine often view their condition as a personal problem, rather than seeking workplace accommodation, due to internalised stigma [29]. Healthcare professionals can play a vital role in advocating for these workplace adaptations and empowering patients with information and self-determination messages. These messages have proven more effective in reducing stigma than messages of pity, as seen in other stigmatised conditions like psychiatric disorders [103].

## 10. Comprehensive Strategies to Reduce Migraine Stigma

Reducing migraine stigma requires a strategic approach that addresses public, structural and internalised stigma through targeted advocacy efforts. Migraine stigma operates at multiple levels, including the general population, healthcare institutions and individual patients [29,82,110,112]. To mitigate this stigma, it is crucial to address each level with evidence-based strategies, encompassing education, contact and sometimes protest [29,112,113,114]. By simultaneously addressing these areas, advocacy can help create a more inclusive environment that recognises and supports individuals with migraines, thereby improving their overall quality of life and diminishing the stigma associated with their condition. A comprehensive approach will help normalise migraine, promote understanding and empower those affected to seek support without fear of judgement or discrimination.

### 10.1. Addressing Public Stigma

Public stigma can be reduced through comprehensive educational campaigns aimed at reshaping societal perceptions of migraines. These campaigns should aim to correct misconceptions and promote an accurate understanding of the condition’s complexity and severity. Educational programmes, which have proven effective in other contexts, should emphasise that migraine is not merely a headache but a multifaceted neurological disorder. By explaining the causes, prevalence, symptoms and treatments available for migraine, these programmes can help replace harmful stereotypes with factual information. For instance, they can highlight the wide spectrum of symptoms experienced during a migraine attack, such as photophobia, sonophobia, nausea, vomiting, dizziness and cognitive or emotional impairment [82,89,108]. These programmes can help promote a more informed and supportive environment for those affected.

Various media channels, such as books, public service announcements, websites, blogs and documentaries, can be leveraged to disseminate these educational messages. Additionally, community-based activities such as walks, races and education camps can make migraine a more visible illness, thereby reducing stigma and helping raise funds for migraine research [109]. However, it is crucial to monitor the outcomes of these educational initiatives to ensure their effectiveness. While these programmes are cost-effective and have been shown to increase workplace productivity and reduce absences by half [82,89,108], it is important to avoid protest efforts that could inadvertently reinforce stereotypes through a rebound effect [103].

Expanding migraine education among healthcare providers is also critical. Increasing the number of headache specialists and educating primary care doctors, neurology residents and fellows can help mitigate negative societal attitudes towards people with migraine [72].

### 10.2. Mitigating Structural Stigma

Structural stigma involves systemic barriers, including inadequate policies and regulations that hinder treatment access and accommodations for individuals with migraines. Advocacy in this area should focus on supportive policies and legislation that facilitate access to necessary treatments and accommodations. For instance, clear guidelines on sick leave and disability benefits for migraine sufferers are essential to ensure that they receive the support they need without facing undue hardship. In countries like the USA, migraine is recognised as a disability, allowing individuals to access workplace accommodations such as flexible schedules and adjustments to their work environment to minimise triggers [82].

The media also plays a significant role in perpetuating negative stereotypes [26]. Inaccurate portrayals in conventional media and social networks often trivialise the condition, reinforcing the stigma surrounding it. For example, a 2018 fashion magazine editor suggested that women could look glamorous by striking the ‘migraine pose’, reinforcing harmful stereotypes and neglecting the condition’s debilitating nature [112]. This type of coverage implies that migraine primarily affects affluent white women who can afford to rest, marginalising others who may be affected. Moreover, typical media depictions of migraine attacks are often slightly realistic at best, contributing to the perpetuation of stereotypes [115,116,117].

Given this, future migraine advocacy should ensure that media portrayals accurately reflect the diverse experiences of individuals with migraines. Moreover, legislative changes are necessary to recognise migraine-related disability and provide access to financial support when required. This is vital to challenge the prevailing social perception that blames individuals for their inability to work due to migraine rather than addressing the underlying social and structural issues [112].

New anti-CGRP therapies have recently been introduced for both acute migraine attacks and prevention, and recent studies have demonstrated that they are more effective and better tolerated than traditional therapeutic options [118,119]. However, their high cost limits their accessibility in many countries [120]. When considering the broader economic impact, including indirect costs such as absenteeism and productivity loss, these therapies have proven to be cost-effective [121,122]. This raises the question of whether the economic arguments against them may reflect a structural stigma towards migraine, affecting their prioritisation in treatment guidelines. Major scientific societies advocate for anti-CGRP monoclonal antibodies as first-line options, highlighting the need for their inclusion despite cost considerations [123,124].

### 10.3. Combating Internalised Stigma

Internalised stigma occurs when individuals with migraines internalise negative societal attitudes, resulting in feelings of shame and self-blame. Advocacy should focus on providing support networks and counselling services that empower individuals to recognise the legitimacy of their experiences and seek help without fear of judgement. Peer support groups and mental health resources play a crucial role in helping individuals build resilience and develop strategies to manage their condition effectively [82,110,114].

The language used in medical practice is also crucial in shaping patient experiences. Using patient-centred language, such as ‘person with migraine disease’ instead of ‘migraineur’ can help avoid defining individuals by their condition [112]. Similarly, replacing terms like ‘medication overuse’ with ‘rebound headache’ can prevent patients from feeling judged by their healthcare providers [112].

## 11. Future Research

Further research is required to assess the impact of stigma on migraine patients and identify the key factors contributing to the stigmatisation of this condition. Research on psychiatric disorders has demonstrated the effectiveness of anti-stigma interventions [124,125]. Whether similar strategies in migraine care can lead to significant improvements in patient management, disability reduction and quality of life is another research imperative. Additionally, research is needed to determine if stigma negatively impacts therapeutic adherence in migraine patients, as seen in psychiatric conditions [126]. The effect of stigma on children with migraine also requires focused study, as early anti-stigma intervention may help prevent long-term adverse outcomes. The advent of anti-CGRP therapies has transformed migraine treatment and likely increased research in this area. Therefore, data on funding migraine research should be updated. Future research should also investigate whether stigma is reduced in countries where migraine care is prioritised and explore with more detail the influence of cultural factors on the stigma associated with migraine. The level of care may influence the presence of stigma and its impact. It is important to know if specialised headache units may help reduce internalised stigma for individuals with migraine by providing accurate diagnosis, education and targeted treatment.

## 12. Discussion

Unravelling migraine stigma is crucial, as it profoundly impacts patients’ lives by influencing their social interactions, family dynamics and professional experiences [29,48]. Rooted in ignorance and misunderstanding, this stigma exacerbates the challenges faced by migraine sufferers. A comprehensive approach that addresses both the neurological aspects and societal perceptions of migraine is essential to improve the quality of life for these patients. Ultimately, tackling stigma should be a primary objective within healthcare interventions, aiming to integrate both clinical treatment and stigma reduction efforts for holistic patient care [29,49,82,127,128,129].

### 12.1. Moving beyond Stereotypes: The Person beyond the Disease

The individual with migraine is much more than their disease. However, societal and medical stereotypes often reduce them to their condition, perpetuating harmful misconceptions [28,29,130]. Historically, there has been a negative stereotype that migraine primarily affects women, which can be considered stigmatising [23,31]. While no study to date has consistently identified significant sex differences in the stigma experienced by patients [5,25,26], the persistent perception of migraine as a ‘women’s disease’ may paradoxically increase migraine-related stigma among men. Another common stereotype is that the disease has a psychiatric origin, due to the frequent confusion of migraine with its common psychiatric comorbidities, such as depression or anxiety [31,32,131]. This mislabelling trivialises the severity of migraine and contributes to the social and medical marginalisation of those affected. As a result, treatment often focuses on psychiatric aspects rather than addressing the neurological nature of the condition.

On the other hand, understanding that not all headaches are the same is crucial. A pervasive stereotype is the belief that ‘everyone gets headaches’, leading to the misconception that all headaches are the same and that migraines are no different from tension-type headaches [132]. This misunderstanding not only fosters stigma but also results in inadequate treatment and support for those suffering from migraines.

Many of these stereotypes have been perpetuated by the scientific literature from past decades [31,32]. To combat this, leaders in neurology must actively highlight the evolving understanding of the causes of migraine and disprove these stereotypes based on scientific evidence [133,134]. By advocating for these advances, they can shift the focus from stigma to empathy, ensuring that patients’ individual needs are prioritised in their care and that patients are not labelled [135,136]. In this regard, language can be a powerful tool, and careful consideration of language use (both in communication with the general public and in the scientific literature) is essential [112,137]. For example, the term ‘migraineur’ can be considered pejorative, stigmatising and labelling individuals by their disease. Ultimately, addressing these issues—gender stereotypes, psychiatric mislabelling, the misunderstanding of headache types and language—is essential in dismantling stigma, recognising the individual beyond their condition and improving the well-being of those affected by migraine.

### 12.2. Educating the Public and Healthcare Professionals: Migraine as a Neurological Disorder

Reducing stigma associated with migraine requires a concerted effort to educate both the general public and healthcare professionals [138]. For the general public, it is essential to provide access to accurate, up-to-date and evidence-based information, particularly because misinformation—often found on social media—can perpetuate stigmatisation [26,115,116,117]. Public education should aim to dispel misconceptions and foster a more supportive and empathetic understanding of the condition.

The education of healthcare professionals is even more critical. Physicians must be fully aware of how profoundly migraine impacts patients’ lives, and, to provide effective care, they need to stay informed about advancements in migraine pathophysiology and the availability of new treatments. Historically, headache has not been the most prestigious subspecialty within neurology, but the advent of new treatments grounded in physiological mechanisms, such as the discovery of calcitonin gene-related peptide (CGRP) and neuroimaging studies showing brain alterations during migraine attacks, are helping to elevate interest in this field [139,140,141,142].

Amplifying patient voices is crucial, as their testimonies offer invaluable insights into the disabling nature of migraine. This perspective is key not only for educating the general public but also for informing healthcare professionals. Understanding these lived experiences can help dismantle prejudices rooted in fear and ignorance, ultimately improving the quality of life for those affected [143,144].

### 12.3. Understanding Migraine Symptoms and Disability

Individuals experiencing migraine-related stigma often report higher rates of disability. Educating both the public and healthcare providers about the diverse symptoms of migraine, including the appearance of prodromal symptoms and interictal symptoms—the period between attacks—is crucial. People with infrequent episodic migraine or without interictal symptomatology may develop stigmatising attitudes toward those with chronic migraine or with multiple symptoms outside of acute attacks, as they may fail to understand the differences and might assume that individuals with more severe conditions are ‘doing something wrong’ or complaining about issues that do not seem related to migraine [110].

Understanding the impact of migraine is further complicated by the interictal burden. If there is already a lack of understanding regarding the disability during the pain phase, it is even more difficult for others to comprehend the interictal symptoms. During this period, patients are often expected to feel completely well and function normally. However, lingering symptoms, along with anxiety and fear of making plans or commitments, can significantly disrupt their daily lives. Support from healthcare providers, families, friends, colleagues and employers is essential in reducing stigma [12,82]. Recognising the inherent disability of migraine is crucial in alleviating the emotional burden of stigma and improving patients’ quality of life, ensuring that their condition does not define their worth or capabilities [7].

### 12.4. Facilitating Life for Migraine Patients: Workplace Accommodations and Social Protection

Education and understanding must be coupled with practical changes to support migraine patients effectively. Misconceptions, such as viewing migraine as an excuse to avoid work or social obligations, contribute to the internalisation of stigma, further compromising well-being. Just as accommodations are made for other chronic conditions, workplaces should offer flexible hours, remote work options and a migraine-friendly environment by minimising triggers like bright lights and loud noises [70]. Implementing these changes not only provides tangible support but also helps patients feel understood and validated in their struggles [95]. Additionally, improving the social protection of migraine patients is essential. Advocacy is needed to drive changes in labour laws and policies, ensuring that migraine sufferers are protected from discrimination and have access to necessary accommodations. Legislative changes should recognise the chronic disabling nature of migraine, offering patients the legal protections they need in the workplace. Enforcing laws that prohibit denying jobs or services to individuals with health issues like migraines is critical for enhancing their quality of life. Compliance with such laws ensures that patients receive the support they deserve, helping them feel acknowledged and respected within their professional and social environments [112].

### 12.5. Prioritising Migraine in Healthcare Systems

Migraine, despite being one of the most prevalent neurological disorders, still remains underprioritised in both medical education and within the field of neurology, leading to delays in diagnosis and treatment that often worsen the condition’s prognosis [144,145]. A key contributing factor to this issue may be the limited focus on migraine in medical schools, where an average of no more than four hours is dedicated to headache training at the undergraduate level [54]. This lack of prioritisation is also evident within the field of neurology itself, where healthcare systems should establish a clear and efficient care pathway that prioritises the rapid diagnosis and treatment of migraine, and increase the number of specialised units, as is conducted with other neurological conditions [146]. Enhancing migraine education in medical schools and continuing professional development programs is essential to ensure timely effective care and to reduce the long-term impact of poorly managed migraine, which is associated with a higher risk of chronification, ultimately improving patients’ overall well-being [75,147].

### 12.6. Combating Isolation and Promoting Support

Living with migraine, particularly when it becomes chronic, can be an isolating experience, worsened by a lack of understanding and support from others. Isolation often leads to loneliness, which is associated with an increased risk of vascular factors, obesity, poor sleep and dietary habits, psychiatric comorbidities and substance abuse—all factors that contribute to migraine chronification [148,149]. Although migraine is not curable, patients can be greatly helped and their quality of life improved with proper management. Healthcare providers and support networks must also offer consistent empathy, support and understanding [82]. This ongoing support significantly helps patients navigate the challenges of living with a chronic condition. Healthcare providers should actively offer both medical treatment and emotional support, creating a therapeutic environment where patients feel heard and understood. Additionally, support groups and patient advocacy organisations are essential in providing a sense of community and shared experience, reducing the isolation that many migraine sufferers feel [110]. By promoting these support systems, we can improve overall well-being and help migraine patients manage both the physical and emotional aspects of their condition [150,151,152].

### 12.7. Strengths, Weaknesses and Clinical Implications

One of the primary strengths of this review is its focus on the often-overlooked issue of stigma associated with migraine. Few studies delve deeply into the multifaceted stigma surrounding this condition, making this review a valuable contribution to the literature. However, a significant limitation is that this is a narrative review, which means that it did not involve a systematic search of the literature, potentially leaving out important studies or perspectives.

Clinically, the implications of this work are clear: addressing stigma should be a priority in improving the care and quality of life for patients with migraine. By bringing greater attention to the stigma surrounding migraine, healthcare providers can better support their patients, leading to more comprehensive care and potentially better outcomes.

## 13. Conclusions

Migraine-related stigma has a profound impact on patients’ lives, affecting their social, family and work relationships and often worsening their condition. Comprehensive destigmatisation strategies are essential to combat this. The key interventions include public and patient education, improved clinician training and workplace adjustments. Additionally, changes in laws, political prioritisation and increased research funding for migraine can help reduce structural stigma. These changes will help patients feel more understood and reduce their internalised stigma. Implementing these measures can significantly improve disease management and enhance the quality of life of individuals with migraine.

## Figures and Tables

**Table 1:** The different types of stigmas in migraine.

Type of Stigma	Description	Examples	Impact Areas
Public Stigma	Stereotypes recognised by the public, including negative or discriminatory attitudes towards individuals with migraines.	Comments like ‘It’s just a headache’, ‘Improve lifestyle habits’ or ‘A typical excuse to skip work’.	Friends, teachers, healthcare providers, employers, coworkers.
Structural Stigma	Occurs when public stigma influences political, legal or organisational policies that limit opportunities for individuals with migraines.	Inadequate research funding for migraines, difficulty obtaining sick leave or disability benefits.	Law, political, government policies, research funding.
Internalised Stigma	Happens when individuals with migraines internalise negative stereotypes, leading to self-blame and decreased self-esteem.	Patients perceiving themselves as ‘incompetent’ or ‘unable to assume responsibilities at work’.	Self-esteem, self-perception.

## Data Availability

No new data were created.
